# Supplementary material for: Clonal selection confers distinct evolutionary trajectories in BRAF-driven cancers
Source: Nat Commun. 2019 Nov 13;10:5143. doi: 10.1038/s41467-019-13161-x (PMC6853924; doi:10.1038/s41467-019-13161-x)
Supplement: Supplementary file 1 — Supplementary Information [file 41467_2019_13161_MOESM1_ESM.pdf]

Supplementary Information

Gopal *et al.*

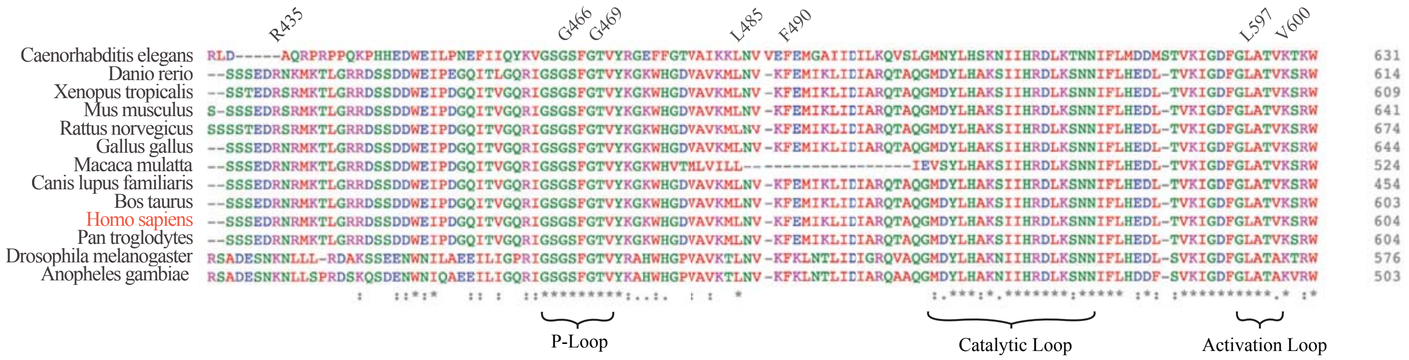

Supplementary Fig. 1. Conservation of BRAF across species. Clustal alignment of BRAF demonstrates highly conserved residues in the kinase domain across 13 species. Residues that correspond to variants profiled in main text Fig. 1f are shown.

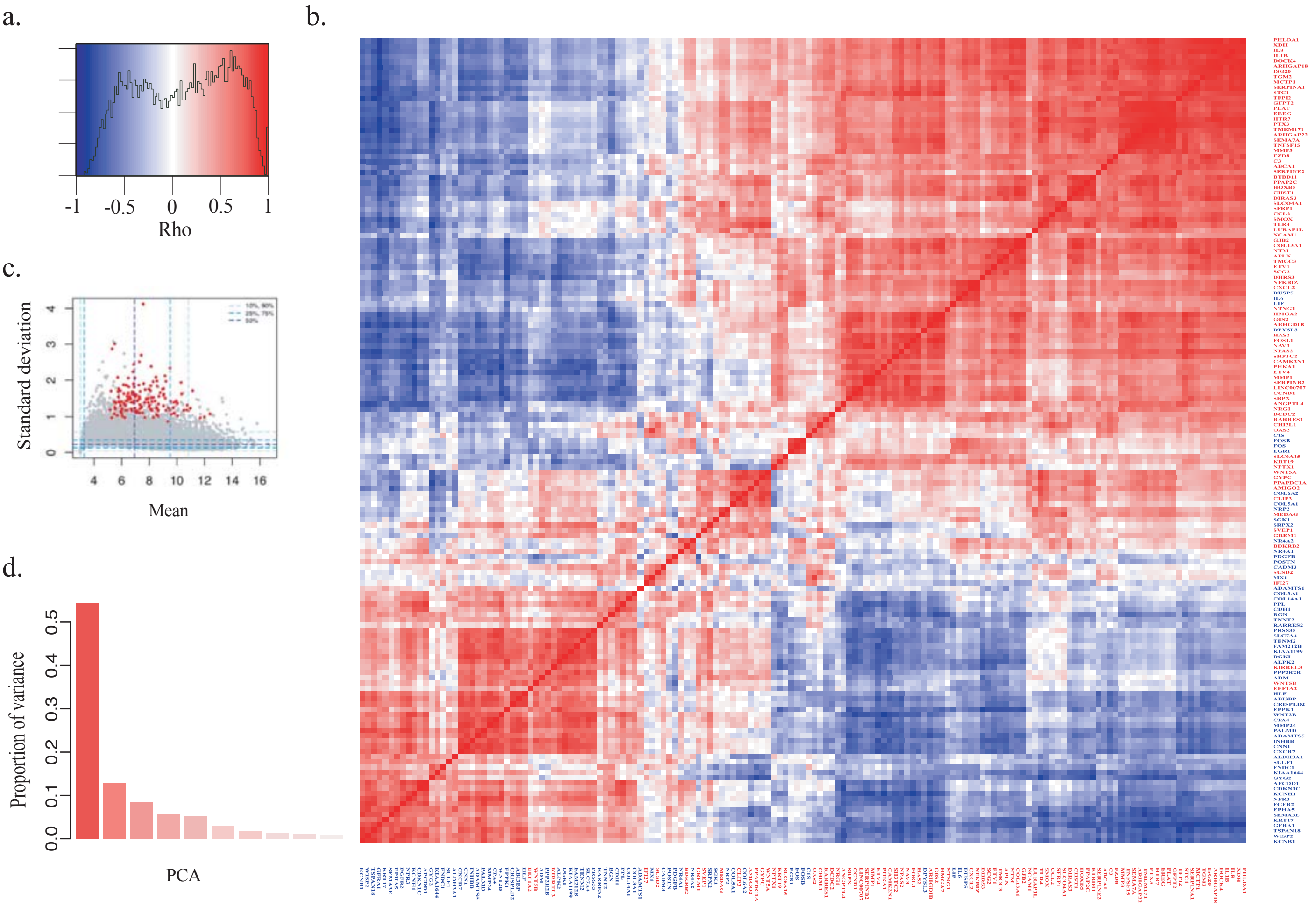

Supplementary Fig. 2. Quality assessment of *BRAF* signature. Kernel density estimates (a) and heatmap (b) of *BRAF* signature genes. Autocorrelation of gene expression across gene indicates signature compactness. (c) Mean and standard deviation of gene expression. *BRAF* signature gene (red) have higher standard deviation and more variable mean expression. (d) Principal component analysis (PCA) plotted against proportion of variance. The *BRAF* gene signature captures most of the variability that exists across the queried samples.

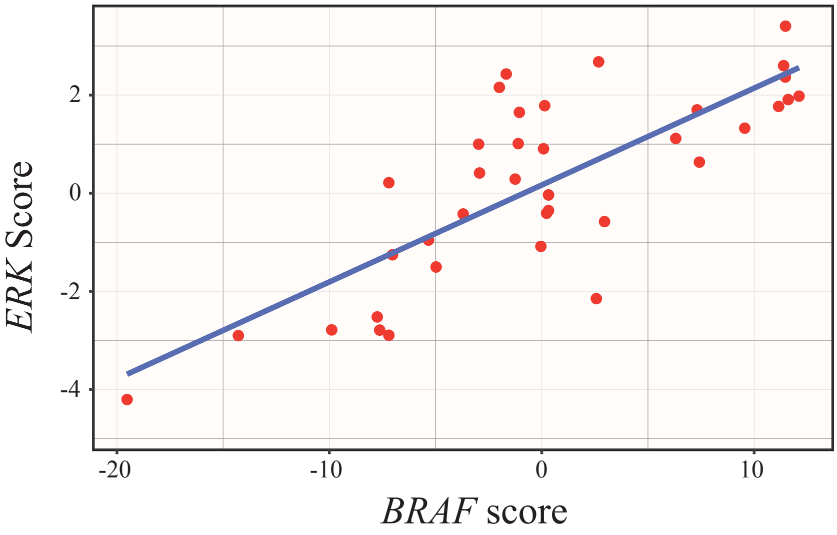

Supplementary Fig. 3. *BRAF* signature score correlated with ERK pathway activity. A signature that estimates ERK activity on the basis of the BIOCARTA_ERK_PATHWAY was highly correlated with the *BRAF* signature score.

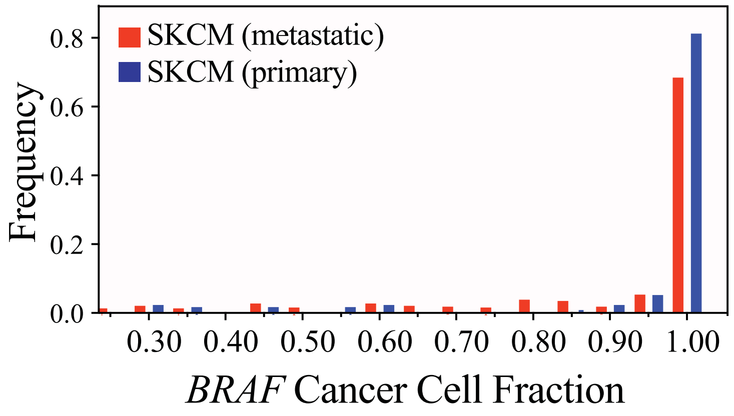

Supplementary Fig. 4. Clonal fixation preceded metastases. Relative frequency of *BRAF* variant CCF values across SKCM tumor sites (primary versus metastatic) suggests that clonal fixation precedes metastatic spread. n = 43 and140 primary and metastatic tumors, respectively.

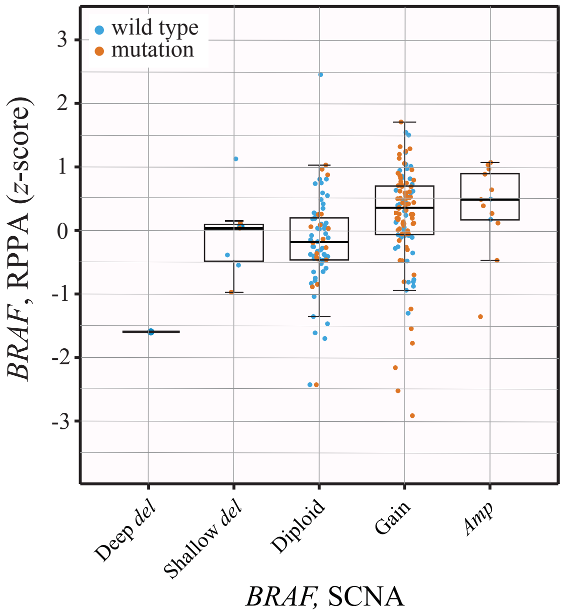

Supplementary Fig. 5. *BRAF* SCNA and protein levels are highly associated. BRAF protein was measured by reverse phase protein array (RPPA) and SCNA were estimated by GISTIC from SKCM tumor samples profiled by TCGA (https://tcga-data.nci.nih.gov/tcga/) and analyzed by (cbioportal.org). Box-plots show the median, the inter-quartile range, and the minimum/maximum after excluding potential outliers.

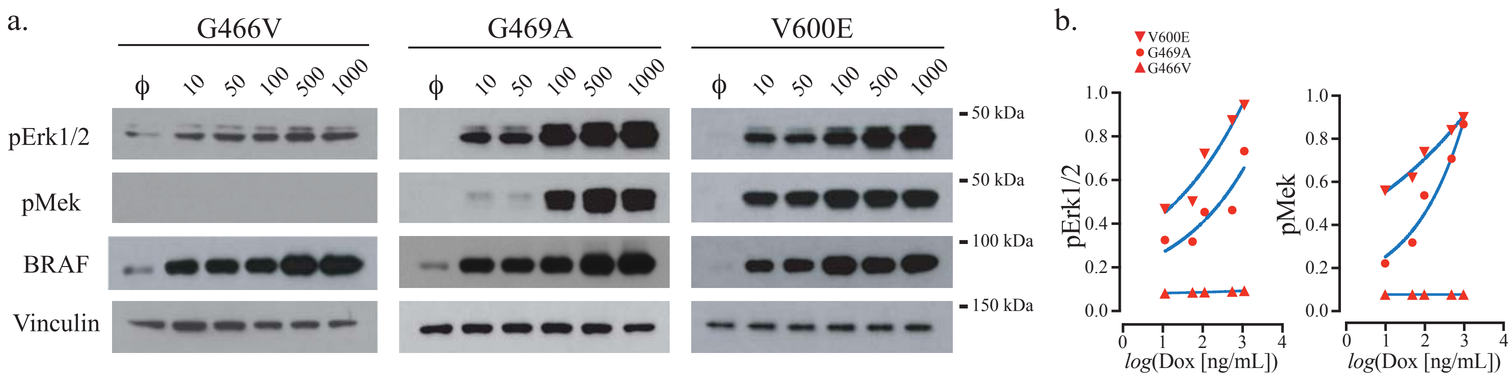

Supplementary Fig. 6. BRAF variants have distinct dynamic ranges of pathway activity. (a) BEAS-2B cells, engineered to express BRAF G466V, G469A, or V600E under a Dox-inducible promoter, were treated with Dox (ng/ml) to determine the effect on signaling. Representative immunoblots are shown 48 hours after induction. (b) Quantification of immunoblots in (a). The *y*-axes were re-scaled from 0 to 1.

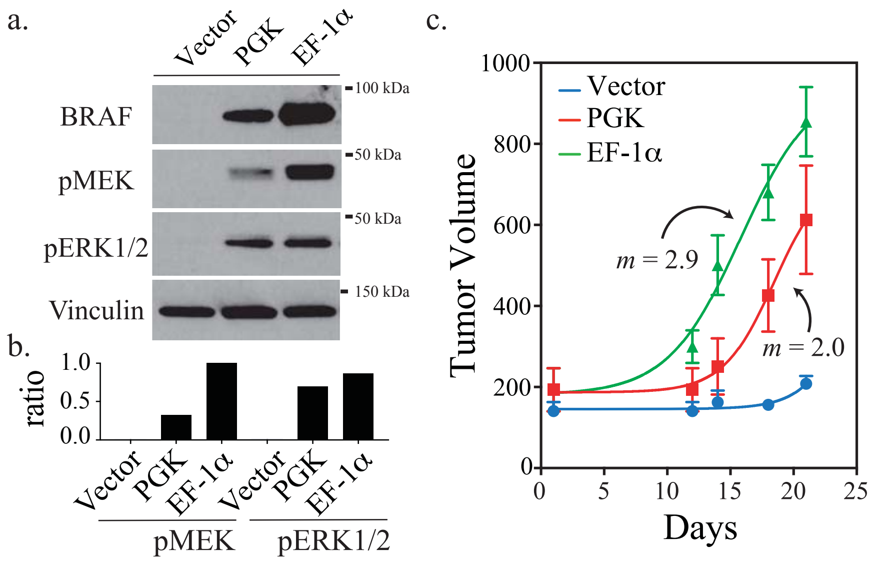

Supplementary Fig. 7. Selection advantage conferred by increasing the levels of *BRAF*^{V600E}. (a) Representative immunblots of BEAS-2B cells, engineered to express BRAF V600E under a PGK or EF-1α expressing promoter. (b) Quantification of immunoblots in (a). (c) BEAS-2B cells expressing BRAF V600E under the respective promoters were injected into the flank of NSG mice. Tumor volumes were measured at least twice weekly. Data represent the mean ± s.e.m; n = 5 independent animals for each condition. Solid lines represent the interpolation of mean using an Boltzmann sigmoidal fit. Slopes estimates from the interpolation are shown.

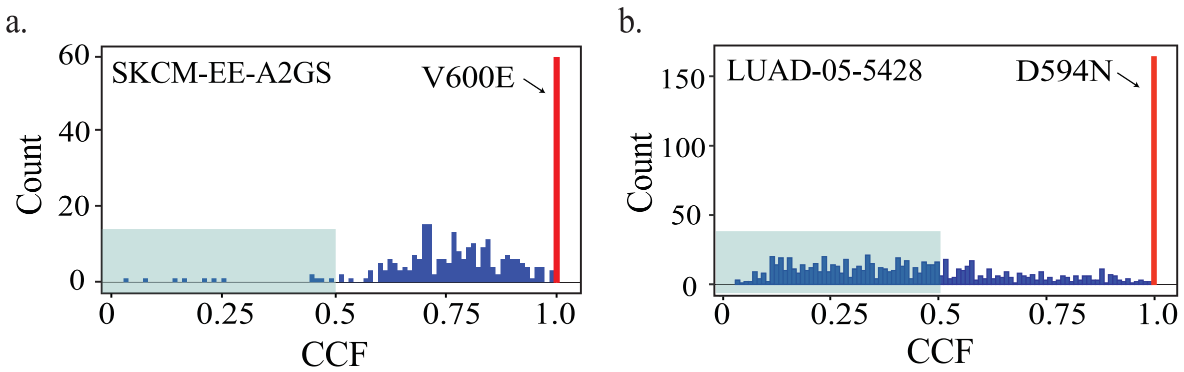

Supplementary Fig. 8. Variant allele fraction distributions are associated with *BRAF* variant multiplicity. Allelic fractions for mutations in two representative tumors, (a) SKCM and (b) LUAD, were rescaled to estimates of cancer cell fraction (CCF) by correcting for sample purity and local copy number. The position of the *BRAF* variant CCF is designated in red. The shaded area highlights regions of the histogram with lower CCF. Mutation numbers in this region were observed to be lower in tumors with higher *BRAF* multiplicity with or without *BRAF* variant fixation (clonality).

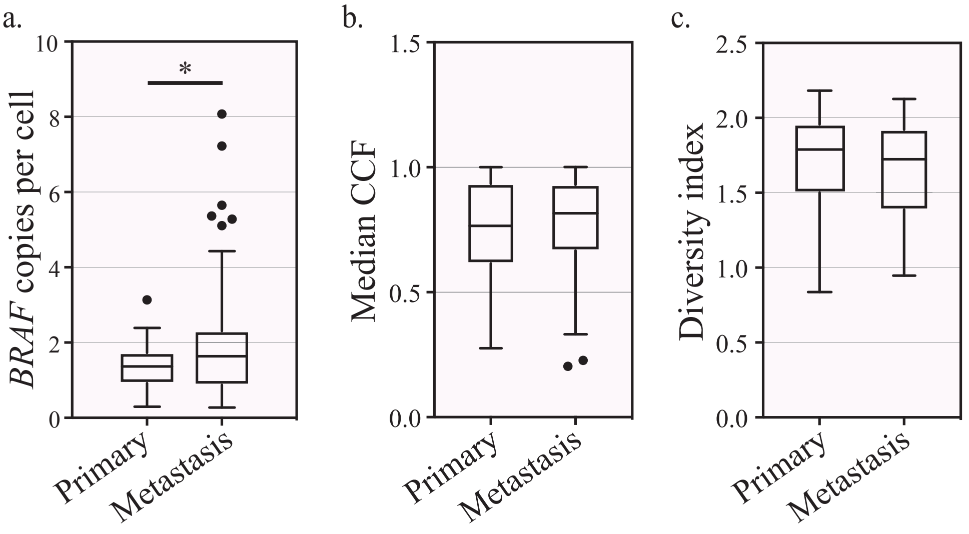

Supplementary Fig. 9. *BRAF* variant copies per cell, median CCF and genetic diversity values in SKCM tumors. Tumors were grouped based on tumor site. Box-plots show the median, the inter-quartile range, and the minimum/maximum after excluding potential outliers. The *p*-values of Welch's t-test comparing the means were < 0.01, 0.42, 0.49 for (a), (b) and (c), respectively. Despite modestly higher variant copies per cell in metastatic tumors, the median and diversity indices were not significantly different in primary *v.* metastatic tumors.

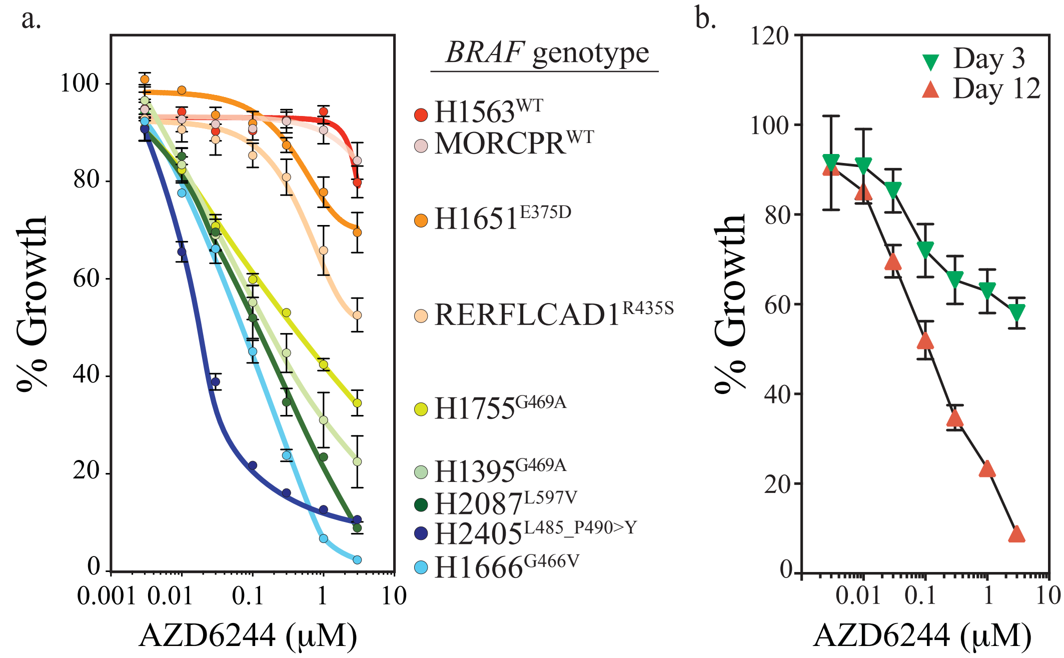

Supplementary Fig. 10. MEK1/2 inhibition effects slow growth in LUAD cells with hypermorphic *BRAF* mutations. (a) MEKi partially decreased the viability of cells with non-V600 BRAF mutations. Cells were treated with AZD6244 (MEKi) and assayed for cellular viability after 12 days. (b) AZD6244 decreases cellular viability overtime, suggesting a growth inhibitory effect. Cellular viability after 72 hours was higher compared to 12 days for H2087 cells containing BRAF L597V. Data are expressed as percent viability relative to vehicle-treated cells and represent the mean ± s.e.m. of at least three independent experiments.

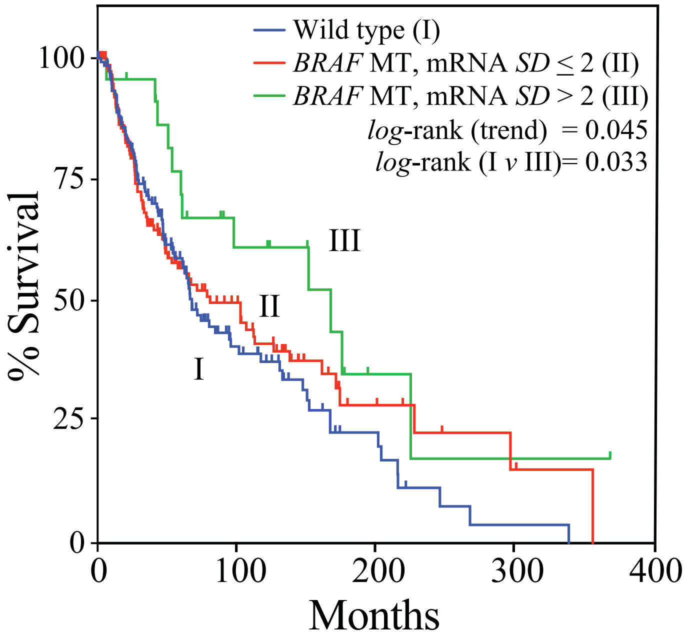

Supplementary Fig. 11. *BRAF* mutations with high *BRAF* mRNA have improved overall survival. Kaplan-Meier survival analysis curve calculated from SKCM patients from TCGA (https://tcga-data.nci.nih.gov/tcga/) separated by *BRAF* mutation and mRNA expression.

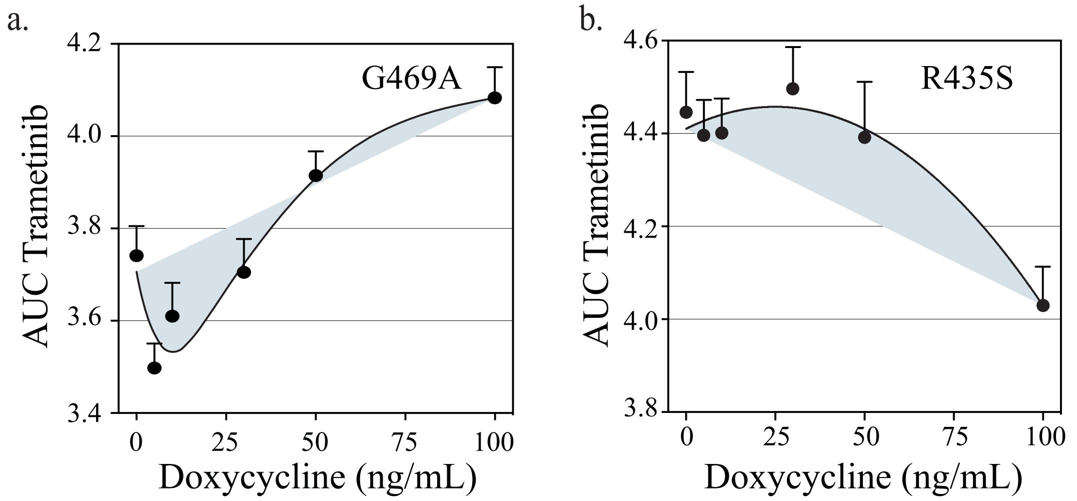

Supplementary Fig. 12. Optimal *BRAF* variant gene dose and drug response varies on the basis of variant identity. Cells expressing (a) G469A or (b) R435S and vector alone were mixed in equal proportion, induced with Dox for 48 hours, and then treated with trametinib (MEKi). Cellular survival was measured 5 days after drug treatment. Data are expressed as the area under the curve (AUC), represent the mean ± s.d., and are representative of at least two independent experiments.

a.

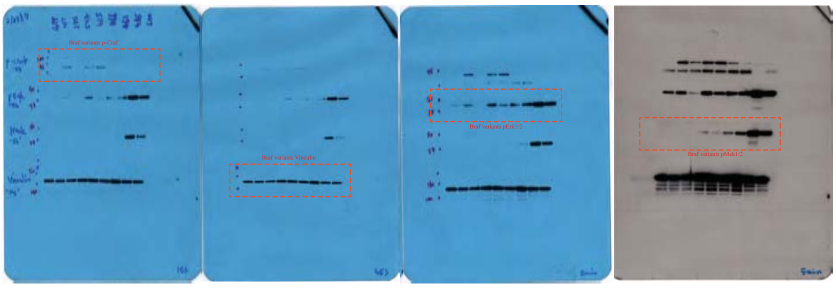

c.

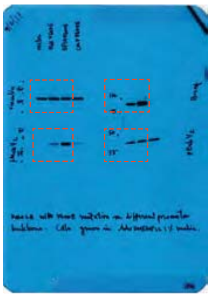

b.

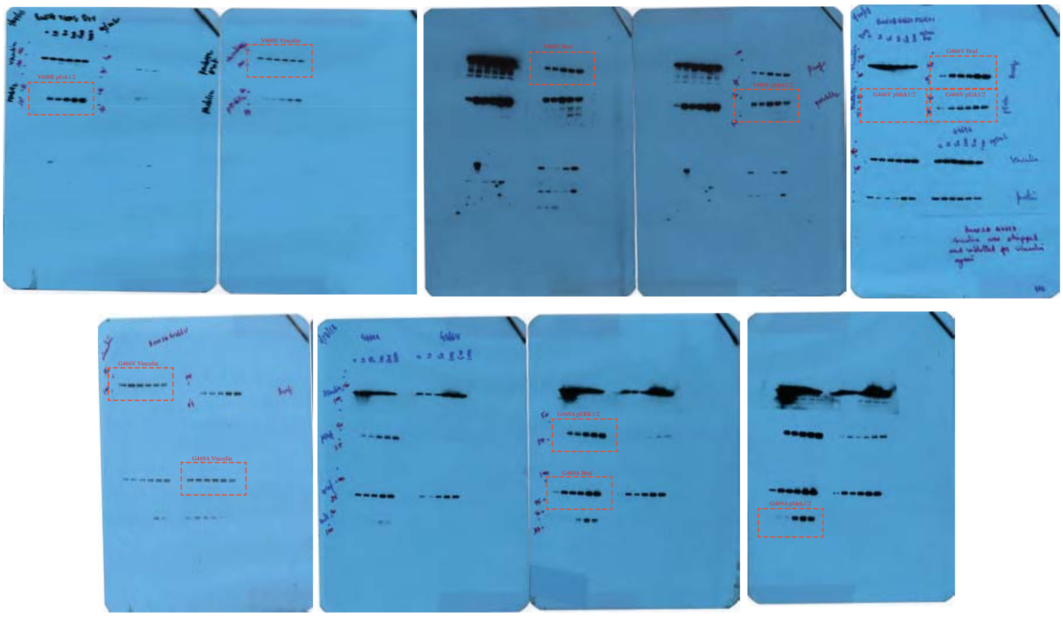

Supplementary Fig. 13. Full images of cropped blots presented in other figures. (a) Full blots for Figure 6c. (b) Full blots for Supplementary Figure 6. (c) Full blots for Supplementary Figure 7.

Supplementary Table 1. BRAF Signature and MSigDB Gene Set Overlap.

Gene Set Name [# Genes (K)]	Description	# Genes in Overlap (k)	*P* value	FDR, Q value
**EPITHELIAL MESENCHYMAL TRANSITION [200]**	Genes defining epithelial-mesenchymal transition, as in wound healing, fibrosis and metastasis.	21	9.05 e^{-26}	2.26 e^{-24}
**TNF-α SIGNALING via NF-κB [200]**	Genes regulated by NF-kB in response to TNF [GeneID=7124].	21	9.05 e^{-26}	2.26 e^{-24}
**HYPOXIA [200]**	Genes up-regulated in response to low oxygen levels (hypoxia).	14	4.87 e^{-15}	8.12 e^{-14}
**KRAS SIGNALING UP [200]**	Genes up-regulated by KRAS activation.	11	6.11 e^{-11}	7.64 e^{-10}
**COAGULATION [138]**	Genes encoding components of blood coagulation system; also up-regulated in platelets.	9	7.85 e^{-10}	7.85 e^{-9}
**COMPLEMENT [200]**	Genes encoding components of the complement system, which is part of the innate immune system.	10	1.18 e^{-9}	8.4 e^{-9}
**INFLAMMATORY RESPONSE [200]**	Genes defining inflammatory response.	10	1.18 e^{-9}	8.4 e^{-9}
**ESTROGEN RESPONSE LATE [200]**	Genes defining late response to estrogen.	9	2.03 e^{-8}	1.27 e^{-7}
**ESTROGEN RESPONSE EARLY [200]**	Genes defining early response to estrogen.	8	3.13 e^{-7}	1.74 e^{-6}
**UV RESPONSE UP [158]**	Genes up-regulated in response to ultraviolet (UV) radiation.	7	8.85 e^{-7}	4.42 e^{-6}
**APOPTOSIS [161]**	Genes mediating programmed cell death (apoptosis) by activation of caspases.	7	1 e^{-6}	4.56 e^{-6}

Supplementary Table 2. Patient-Derived Tumor Xenograft Models Using in the Study.

PDX ID	CBX389	CCX124	CBX443
**Age**	74	66	69
**Gender**	female	female	female
**Diagnosis**	LUAD	LUAD	SKCM
**Prior treatment**	Carboplatin + Alimta; Nivolumab	Carboplatin + Pemetrexed	naïve
**Response to prior treatment**	Progression	Progression	NA
**Primary, metastasis, recurrence**	Primary	Metastasis	Metastasis
**Organ from which specimen obtained**	Lung	Liver	Lung
**Tissue histology**	adenocarcinoma	adenocarcinoma	melanoma
**Disease stage; classification**	T3N1M0 (IIIA)	T2N2M1b (IVB)	T4bN3M1c (IV)
**Genotype**	BRAF G469A	BRAF V600E	BRAF V600G
**Sample type**	Biopsy	Biopsy	Pleural Effusion
**Mouse strain and source**	NOD.Cg-PrkdcscidIl2rgtm1Wjl/SzJ	NOD.Cg-PrkdcscidIl2rgtm1Wjl/SzJ	NOD.Cg-PrkdcscidIl2rgtm1Wjl/SzJ
**Strain immune system humanized?**	No	No	No
**Tumour preparation**	Suspension	Suspension	Suspension
**Injection type and site**	Subcutaneous, flank	Subcutaneous, flank	Subcutaneous, flank
