# Supplementary material for: Clonal selection confers distinct evolutionary trajectories in BRAF-driven cancers
Source: Nat Commun. 2019 Nov 13;10:5143. doi: 10.1038/s41467-019-13161-x (PMC6853924; doi:10.1038/s41467-019-13161-x)
Supplement: Supplementary file 3 — Description of Additional Supplementary Files [file 41467_2019_13161_MOESM3_ESM.pdf]

Description of Additional Supplementary Files

File Name: Supplementary Data 1
Description: Covariate Associations with BRAF Mutations in SKCM.

File Name: Supplementary Data 2
Description: Primer Sequences and Source Vectors.

File Name: Supplementary Software 1
Description: Source code for readers to independently measure cancer cell fraction and conduct clonal sweep simulations.
