# Supplementary material for: Clonal selection confers distinct evolutionary trajectories in BRAF-driven cancers
Source: Nat Commun. 2019 Nov 13;10:5143. doi: 10.1038/s41467-019-13161-x (PMC6853924; doi:10.1038/s41467-019-13161-x)
Supplement: Supplementary file 7 — Reporting Summary [file 41467_2019_13161_MOESM7_ESM.pdf]

nature research

Corresponding author(s): Mohamed Abazeed, MD, PhD

Last updated by author(s): Mar 1, 2019

Reporting Summary

Nature Research wishes to improve the reproducibility of the work that we publish. This form provides structure for consistency and transparency in reporting. For further information on Nature Research policies, see Authors & Referees and the Editorial Policy Checklist.

Statistics

For all statistical analyses, confirm that the following items are present in the figure legend, table legend, main text, or Methods section.

n/a	Confirmed	
☐	☒	The exact sample size (*n*) for each experimental group/condition, given as a discrete number and unit of measurement
☐	☒	A statement on whether measurements were taken from distinct samples or whether the same sample was measured repeatedly
☐	☒	The statistical test(s) used AND whether they are one- or two-sided<br>*Only common tests should be described solely by name; describe more complex techniques in the Methods section.*
☒	☐	A description of all covariates tested
☐	☒	A description of any assumptions or corrections, such as tests of normality and adjustment for multiple comparisons
☐	☒	A full description of the statistical parameters including central tendency (e.g. means) or other basic estimates (e.g. regression coefficient) AND variation (e.g. standard deviation) or associated estimates of uncertainty (e.g. confidence intervals)
☐	☒	For null hypothesis testing, the test statistic (e.g. *F*, *t*, *r*) with confidence intervals, effect sizes, degrees of freedom and *P* value noted<br>*Give P values as exact values whenever suitable.*
☒	☐	For Bayesian analysis, information on the choice of priors and Markov chain Monte Carlo settings
☐	☒	For hierarchical and complex designs, identification of the appropriate level for tests and full reporting of outcomes
☐	☒	Estimates of effect sizes (e.g. Cohen's *d*, Pearson's *r*), indicating how they were calculated

Our web collection on statistics for biologists contains articles on many of the points above.

Software and code

Policy information about availability of computer code

Data collection	.csv and .txt files were used for data collection.
Data analysis	Cancer cell fraction and selective sweep simulator source code is provided to to the editors/reviewers and will be made available in a community repository upon publication. The cancer cell fraction code includes chunks of source code from other open source repositories as comprehensively annotated in the script files. Other software that is used, namely phyloWGS and sigQC, are publicly available and cited in the manuscript.

For manuscripts utilizing custom algorithms or software that are central to the research but not yet described in published literature, software must be made available to editors/reviewers. We strongly encourage code deposition in a community repository (e.g. GitHub). See the Nature Research guidelines for submitting code & software for further information.

Data

Policy information about availability of data

All manuscripts must include a data availability statement. This statement should provide the following information, where applicable:

- Accession codes, unique identifiers, or web links for publicly available datasets
- A list of figures that have associated raw data
- A description of any restrictions on data availability

Some of the data used in the current study are available through the Genomic Data Commons portal (https://gdc-portal.nci.nih.gov), the Cancer Target Discovery and Development (CTD2) initiative (https://ocg.cancer.gov/programs/ctd2) or the Cancer Cell Line Encyclopedia (http://www.broadinstitute.org/ccle). These datasets are publicly available. Other datasets analyzed during the current study are available from the corresponding author on reasonable request.

Field-specific reporting

Please select the one below that is the best fit for your research. If you are not sure, read the appropriate sections before making your selection.

☒ Life sciences ☐ Behavioural & social sciences ☐ Ecological, evolutionary & environmental sciences

For a reference copy of the document with all sections, see nature.com/documents/nr-reporting-summary-flat.pdf

Life sciences study design

All studies must disclose on these points even when the disclosure is negative.

Sample size	Sample size was determined using the RE/AR model as indicated in the methods.
Data exclusions	No data were excluded form the analyses.
Replication	All reported attempts at replication were successful.
Randomization	Mice were block randomized.
Blinding	Blinding of the investigator was not possible because he/she must identify the mice in the corresponding treatment arm.

Reporting for specific materials, systems and methods

We require information from authors about some types of materials, experimental systems and methods used in many studies. Here, indicate whether each material, system or method listed is relevant to your study. If you are not sure if a list item applies to your research, read the appropriate section before selecting a response.

Materials & experimental systems

n/a	Involved in the study	
☐	☒	Antibodies
☐	☒	Eukaryotic cell lines
☒	☐	Palaeontology
☐	☒	Animals and other organisms
☒	☐	Human research participants
☐	☐	Clinical data

Methods

n/a	Involved in the study	
☒	☐	ChIP-seq
☒	☐	Flow cytometry
☒	☐	MRI-based neuroimaging

Antibodies

Antibodies used	Anti-actin (8H10D10), anti-phospho-MEK1/2 (41G9), anti-phospho-p44/42 MAPK (Erk1/2), anti-phospho-c-Raf (Ser 259) and anti-BRAF (D9T6S) were from Cell Signaling Technology (Beverly, MA). Anti-vinculin Ab-1 (VLN01) was from Thermo-Fisher Scientific (Waltham, MA).
Validation	All antibodies used in this study are both in common use and have been validated by the manufacturer.

Eukaryotic cell lines

Policy information about cell lines

Cell line source(s)	Cancer Cell Line Encyclopedia
Authentication	SNP genotype matching was available for a cancer cell line through the CCLE project.
Mycoplasma contamination	All cultures were tested to ensure absence of Mycoplasma.
Commonly misidentified lines (See ICLAC register)	We cross-referenced our cell lines with the database of cross-contaminated or misidentified cell lines curated by the International Cell Line Authentication Committee and NCBI BioSample and no cell lines that could have been contaminated or misidentified were used in this study.

Animals and other organisms

Policy information about studies involving animals; ARRIVE guidelines recommended for reporting animal research

Laboratory animals	Female NSG mice between the ages of 6-20 weeks were used.
Wild animals	The study did not involve wild animals.
Field-collected samples	The study did not involve samples collected from the field.
Ethics oversight	IACUC

Note that full information on the approval of the study protocol must also be provided in the manuscript.

Clinical data

Policy information about clinical studies

All manuscripts should comply with the ICMJE guidelines for publication of clinical research and a completed CONSORT checklist must be included with all submissions.

Clinical trial registration	Not applicable. Per TCGA. No independent interventional clinical studies were conducted in this study.
Study protocol	Not applicable. Per TCGA. No independent interventional clinical studies were conducted in this study. A tissue repository was used as a source to develop patient-derived xenografts. That protocol is IRB 07-267.
Data collection	Not applicable. Per TCGA. No independent interventional clinical studies were conducted in this study. Patient and tumor characteristics from IRB 07-267 were extracted from the medical charts of patients at the Cleveland Clinic and stored in RedCapp frpm 2013-2018.
Outcomes	Not applicable. Per TCGA. No independent interventional clinical studies were conducted in this study.
